# Genipin-Crosslinked Gelatin/Chitosan-Based Functional Films Incorporated with Rosemary Essential Oil and Quercetin

**DOI:** 10.3390/ma15113769

**Published:** 2022-05-25

**Authors:** Swarup Roy, Jong-Whan Rhim

**Affiliations:** 1 Department of Food and Nutrition, BioNanocomposite Research Institute, Kyung Hee University, 26 Kyungheedae-ro, Dongdaemun-gu, Seoul 02447, Korea; swaruproy2013@gmail.com; 2 School of Bioengineering and Food Technology, Shoolini University, Solan 173229, Himachal Pradesh, India

**Keywords:** chitosan/gelatin, functional film, cross-linking, genipin, rosemary essential oil, quercetin

## Abstract

Functional food packaging films were prepared using a binary mixture of chitosan and gelatin through crosslinking with genipin and hybridization with rosemary essential oil and quercetin. The mixture of chitosan and gelatin produced the compatible film, and the added fillers also showed good compatibility. The physical properties of the chitosan/gelatin film were not greatly affected by crosslinking with genipin, and the functionality of the composite film was increased by the addition of rosemary essential oil and quercetin. The bioactive additives did not significantly affect the hydrophobicity and water vapor barrier properties of the chitosan/gelatin film but significantly changed the color, while the mechanical and thermal properties were slightly affected. The addition of these functional fillers significantly improved the UV protection, antioxidant, and antibacterial properties of the chitosan/gelatin film. Therefore, the novel chitosan/gelatin film with genipin crosslinking and the integration of rosemary essential oil and quercetin is considered to have high potential for applications in active food packaging.

## 1. Introduction

In today’s food packaging field, there is an increasing trend to replace non-degradable plastic packaging materials using annually renewable natural biopolymers to solve the problems of environmental pollution and resource depletion caused by the use of synthetic plastics [1,2]. Biopolymer-based food packaging materials are environmentally friendly, biocompatible, and even edible without any safety concerns [3,4]. The edible films are palatable, protect the packed food, and prolong the shelf-life [3,5,6]. Animal and plant-derived carbohydrates and proteins are mainly used as edible biopolymer packaging materials [7,8,9].

It has recently been shown that polysaccharide-protein mixtures are useful for making biodegradable active packaging films [10,11,12,13,14,15]. For this purpose, chitosan and gelatin are ideal choices for producing binary blends of biopolymer films. Several reports have already been published with promising blends of chitosan and gelatin [16,17,18,19,20]. However, the physical properties of the chitosan/gelatin-based films can be improved through crosslinking between biopolymers. 

Polymer intermolecular crosslinking improves a film’s mechanical and hydrophobic properties and creates integrated complex networks by forming chemical bonds (intramolecular or intermolecular) with molecular polymer chains [21,22,23]. As a low-toxic natural crosslinking agent for both chitosan and gelatin, genipin could be an ideal choice for making crosslinked chitosan/gelatin-based films [24,25]. Genipin is a water-soluble crosslinking agent isolated from gardenia, which reacts with the -NH_2_ group of the polymer to give it a dark blue color [26]. Another interesting fact is that genipin has ~10^4^ times less toxicity than glutaraldehyde [27].

Although the chitosan/gelatin-based film has some functional properties (antimicrobial and antioxidant properties) [28,29], it is not sufficient for application in active packaging, and thus it is necessary to enhance the functionality of the film by adding other functional substances. Recently, essential oils of natural origin have emerged as promising functional materials in active food packaging. 

Rosemary essential oil (RO) is well known for its many beneficial properties, such as anti-inflammatory, antioxidant, antibacterial, analgesic, and detoxifying properties, and is also on the FDA’s GRAS rating [30,31]. RO contains many bioactive functional ingredients, such as terpenes, camphenes, limonene, camphor, borneol, cineol, and linalool. [32]. Rosemary essential oil is commonly used as a food flavoring agent [33]. Thus, RO is an ideal choice for making biopolymer-based functional packaging films. Previously, there were already some reports of RO-containing films for active packaging applications [31,34,35].

On the other hand, the most commonly found dietary bioactive compound quercetin can be another useful biomaterial for active packaging due to its good antioxidant and antibacterial activity [36,37]. Quercetin is a polyphenolic compound commonly found in many fruits and vegetables [38]. Quercetin has been commonly used in the food industry to prevent the oxidation of foods [39]. Due to its excellent functional properties, quercetin has also been used to manufacture various biopolymer-based packaging films [20,36,38,39,40].

It is expected that the functional properties of the film will be improved by mixing RO and quercetin in a genipin-crosslinked chitosan/gelatin-based blend film. Therefore, this study aimed to prepare a functional genipin-crosslinked chitosan/gelatin-based film by adding rosemary essential oil and quercetin.

## 2. Materials and Methods

### Preparation of Films

Chitosan/gelatin-based binary composite films were prepared by mixing chitosan and gelatin at a 1:1 ratio [41]. Chitosan solution (2 wt%) was prepared by dissolving 2 g chitosan in 100 mL of 1% acetic acid solution, and gelatin solution (2 wt%) was prepared by dissolving 2 g gelatin in 90 mL of distilled water with vigorous stirring at 60 °C for 20 min. The chitosan and gelatin solutions were combined, and we added 40 mg (1 wt% of polymers) of genipin as a crosslinker. Three different functional films were prepared by adding quercetin (QCT) and rosemary essential oil (RO) to the genipin-added chitosan/gelatin film solution. 

To prepare the QCT-added film solution, 5 mL (1 wt% of polymer) of quercetin solution prepared in a 1:1 ethanol and water mixture was added to the chitosan/gelatin film solution. To prepare the RO-added film solution, 5 mL (2 wt% of polymer) of rosemary essential oil emulsified using Tween 80 was added to the chitosan/gelatin film solution. For the preparation of the QCT and RO-added film solution, we added 5 mL of quercetin solution and 5 mL of RO solution sequentially to the chitosan/gelatin film solution. The film-forming solution was then cast on a flat Teflon film-coated glass plate and dried at room temperature for 48 h. 

**Scheme 1:**
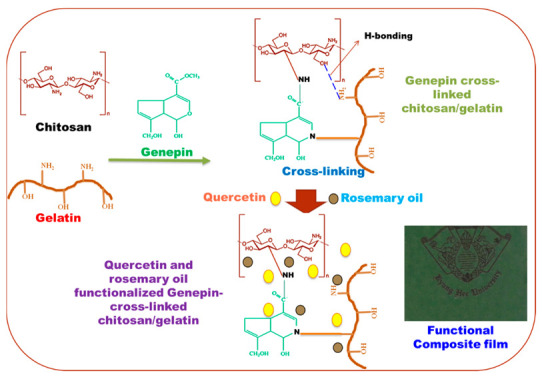
Schematic diagram of the biocomposite film fabrication and interactions between polymers and fillers.

In addition, the neat chitosan/gelatin film without additives and only rosemary oil and quercetin were also prepared using the same procedure. The prepared films were designated CTS/GTN (chitosan/gelatin), CTS/GTN^Gen^, CTS/GTN^Gen^/QCT, CTS/GTN^Gen^/RO, and CTS/GTN^Gen^/QCT/RO films, respectively, depending on their components. CTS, GTN, Gen, QCT, and RO are abbreviations for chitosan, gelatin, genipin, quercetin, and rosemary essential oil. The film fabrication and interactions among the polymer-polymer and fillers and polymers are pictorially shown in Scheme 1. The methods for the film characterization and properties determination are shown in Supplementary Materials.

## 3. Results and Discussion

### 3.1. Properties of the Film

#### 3.1.1. Optical Properties

**Figure 1:**
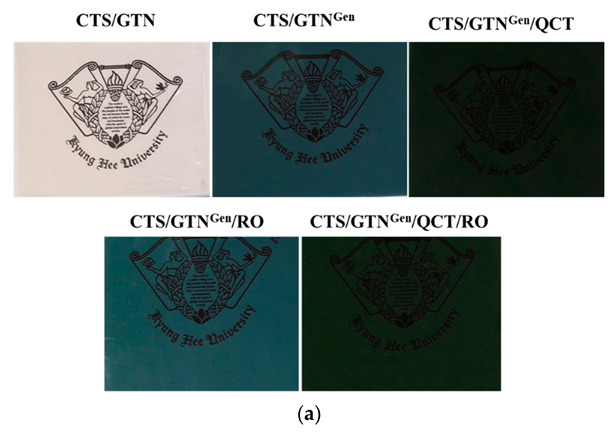

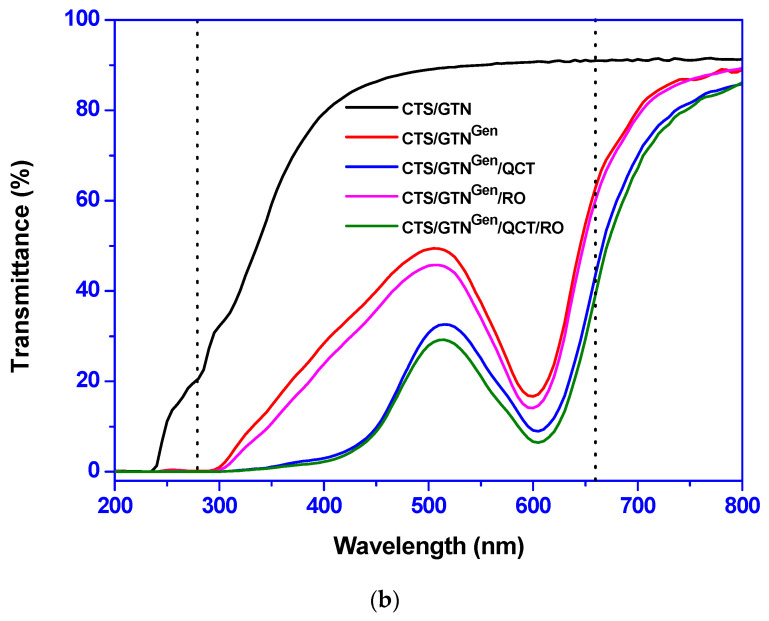
The appearance (**a**) and light transmission spectra (**b**) of the chitosan/gelatin-based films.

The visual appearance of the fabricated films is shown in Figure 1a. The control chitosan/gelatin film was transparent without any color tint; however, the other films were translucent and colored. The addition of genipin made the film blue, while the addition of quercetin to the genipin-crosslinked film developed green color. Although the color of quercetin is yellow, when quercetin is added to the genipin-added chitosan/gelatin film, it is combined with the blue color of the film to give it a green color. However, when rosemary oil was added, the blue color of the film was maintained, and the brightness was slightly increased.

The light transmission spectra of the films are shown in Figure 1b. The neat chitosan/gelatin film did not show any characteristic absorption in the 200–800 nm range. On the other hand, the genipin-added films completely blocked UV light below 300 nm and showed the maximum and minimum light transmittance at 507–517 nm and 600–606 nm, respectively, which were due to the UV blocking properties of genipin and green-blue light absorption at ~510 nm and orange-yellow light absorption at ~600 nm by genipin and quercetin, respectively [25]. 

A similar effect of genipin on UV-visible light absorption properties was observed in the genipin-added sodium caseinate film [24]. The transparency and UV-light barrier of the chitosan/gelatin-based films were evaluated by determining the light transmittance at 660 and 280 nm, respectively, and the results are shown in Table 1. The neat chitosan/gelatin film showed some UV-barrier (T_280_) properties (~21%) with high transparency (T_660_) over 90%. The UV-blocking property of the neat chitosan/gelatin film was attributed to UV absorption by chitosan and gelatin. The addition of the crosslinking agent, genipin, had a great effect on the light transmission of the film, and completely blocked UV-light. When genipin was added, the UV protection effect was greatly improved; however, the transparency of the film was also reduced from 90% to 60%. 

When quercetin was added, the transparency of the film decreased more, whereas when rosemary oil was added, there was no significant effect. The UV-light blocking effect of genipin is consistent with previously reported results [24,25].

The film surface color values are also shown in Table 1. The neat chitosan/gelatin film exhibited high lightness (*L*-value) and slight yellowness (*b*-value) due to the chitosan solution having a yellowish color. The film’s brightness was greatly reduced by adding genipin and further decreased with the addition of rosemary oil and quercetin. The addition of genipin significantly changed the *b*-value due to the development of the blue color in the film. 

Interestingly, the film containing quercetin became green by mixing the blue caused by genipin and the yellow of quercetin, and thus the *a*-value of the film decreased. As expected from the changes in the *L*, *a*, and *b* values of the films, the Δ*E* of all composite films was significantly increased compared to the neat chitosan/gelatin films. Similar color changes were observed in the genipin-added sodium caseinate film [24].

#### 3.1.2. Morphology of the Film

**Figure 2:**
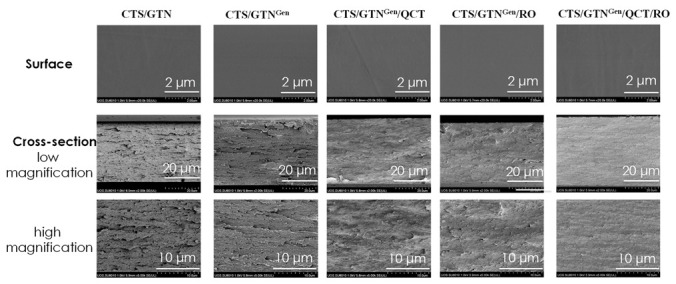
Morphology (surface and cross-section) of the chitosan/gelatin-based films.

The microstructure of the chitosan/gelatin-based films was observed using a field emission scanning microscope (FE-SEM), and the results are shown in Figure 2. The surface image of all films showed the intact and smooth-surfaced, and the added filler materials were homogeneously mixed in the binary polymer film matrix. The cross-section image showed some layer-by-layer structure in the neat chitosan/gelatin film. At the same time, the addition of the crosslinker and bioactive fillers made clear structural modifications with a reduced layer structure in the films. 

It is believed that the mixing of quercetin and rosemary oil created a coating on the layers of the film, possibly as a result of the reduced structured layer. Overall, the SEM images showed that the added filler was compatible with the matrix polymer. Similar results were reported for graphene-oxide-reinforced gelatin films crosslinked with genipin [25].

#### 3.1.3. Thermal Stability of the Film

**Figure 3:**
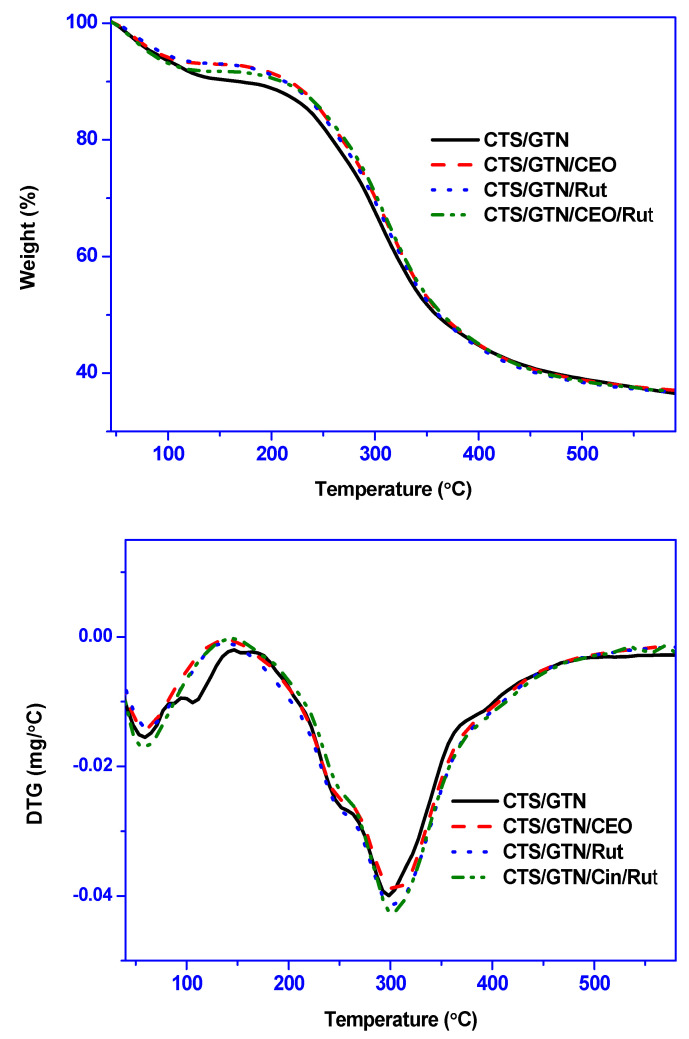
TGA and DTG thermograms of chitosan/gelatin-based films.

The thermal stability of the chitosan/gelatin-based films was evaluated using TGA analysis, and the results are shown in Figure 3. All films showed a two-stage thermal decomposition pattern. The initial weight loss observed at 40–125 °C was due to the evaporation of physisorbed moisture. The major weight change of the film occurred in the range of 150–420 °C, with the highest weight change at around 300 °C, which was due to the decomposition of polymers (chitosan and gelatin) [19,42]. 

Crosslinking the chitosan/gelatin film with genipin increased the maximum degradation temperature (~10 °C). However, the addition of the bioactive functional fillers (quercetin and rosemary essential oil) did not significantly affect the thermal stability of the film. The increased thermal stability of the chitosan/gelatin film by crosslinking with genipin was consistent with the previous findings in the genipin-crosslinked graphene oxide mixed gelatin-based films [25]. As with the present results, it was previously reported that the addition of bioactive ingredients and essential oils did not significantly affect the thermal stability of biopolymer films, such as chitosan/gelatin-based films [19]. 

Table S1 (Supplementary Materials) shows more detailed TGA analysis results. The T_onset_/T_end_ temperature did not change, while theT_0.5_ (50% decomposition) temperatures changed slightly (5–10 °C) in the functional composite films. The residual charcoal content of chitosan/gelatin-based films was about 36–38%, and it was not significantly affected by the addition of crosslinking agent and bioactive fillers. The developed biopolymer-based film had excellent thermal stability; however, its thermal stability is much lower than that of synthetic plastic films, such as LDPE, which have a maximum thermal decomposition temperature of about 450 °C [43].

#### 3.1.4. Mechanical Properties of the Films

The mechanical properties of the chitosan/gelatin-based films are shown in Table 2. The thickness of the films was not significantly changed by adding the crosslinker and bioactive fillers. The chitosan/gelatin film was strong, showing a high tensile strength (TS) of 77.3 MPa. Yadav et al. also found that chitosan blended with gelatin formed a strong film with a maximum breaking strength of 16.1 N; however, we could not compare the TS of the film directly since they did not measure the film thickness [20]. 

The TS of the chitosan/gelatin film was significantly increased through the crosslinking by genipin, and it further increased slightly by the addition of functional fillers (quercetin and rosemary essential oil); however, the increase was not statistically significant. Similar to the case of TS, the stiffness of the films evaluated by elastic modulus (EM) was significantly increased by adding crosslinking agent and functional fillers. 

On the other hand, the elongation at break (EB) of the chitosan/gelatin decreased significantly when the crosslinker and functional fillers were added. The observed mechanical behavior agrees with previously reported genipin-crosslinked sodium casein edible films and chitosan/astaxanthin films [24,26]. Yadav et al. also found that the addition of quercetin to chitosan/gelatin film increased the strength and flexibility of the film [20].

The mechanical strength of the fabricated biopolymer-based film is much higher than a commonly used synthetic plastic film like polyethylene (24 MPa) [44], LDPE (22 MPa) [43], and HDPE (45 MPa) [45]. On the other hand, the flexibility of the biopolymer-based film is much lower than the commercial plastic films, such as polyethylene (785%) [44], LDPE (806%) [43], and HDPE (255%) [45]. Therefore, it is necessary to improve the flexibility of the biopolymer-based film. Biopolymer-based polymers with functional properties can be a promising alternative to solve these problems. Although the trend of replacing plastic packaging materials with biodegradable materials is increasing, many studies are still needed for practical use.

#### 3.1.5. Water Vapor Permeability (WVP) and Water Contact Angle (WCA) of the Film

The water vapor barrier and surface wettability of the chitosan/gelatin-based films were evaluated using WVP and WCA, respectively, and the results are shown in Table 2. The WCA of the film was slightly increased by the addition of crosslinking agent and functional fillers; however, the increase was not significantly different. Although the strength was increased by forming a dense structure of the film by forming crosslinks in the film by genipin, the water vapor barrier properties of the film did not change significantly. 

Previously, similar results were observed in the genipin-crosslinked casein sodium-based film and the chitosan/astaxanthin film [24,26]. The WVP of the film further increased, although it was not significant, by the addition of the functional fillers, which are most likely due to the poor interactions between the fillers and polymer matrix. Similar results were reported for chitosan/gelatin-based films with added essential oils [19]. 

The water vapor barrier properties of the fabricated biopolymer-based film are significantly lower than the commonly used synthetic plastic films, such as polyethylene (1.91  × 10^−12^ g∙m/m^2^∙s∙Pa) [44] and LDPE (0.91 × 10^−12^ g∙m/m^2^∙s∙Pa) [46]. Therefore, although biopolymer-based films may be a promising choice, there is still room for improving the water vapor barrier properties of the films to meet the properties of commercial plastic films.

The WCA of the chitosan/gelatin film was 60.5°, indicating it has a hydrophilic surface. The addition of crosslinking agent and functional fillers did not significantly change the surface hydrophilicity of the film. A similar effect of WCA has been observed in the rutin-added chitosan/poly(vinyl alcohol) and genipin-crosslinked sericin/poly(vinyl alcohol) films [47,48].

### 3.2. Antimicrobial Activity

**Figure 4:**
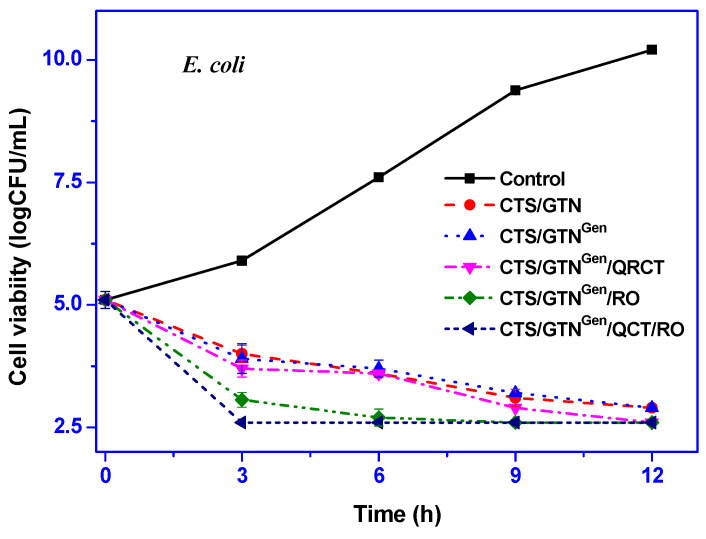

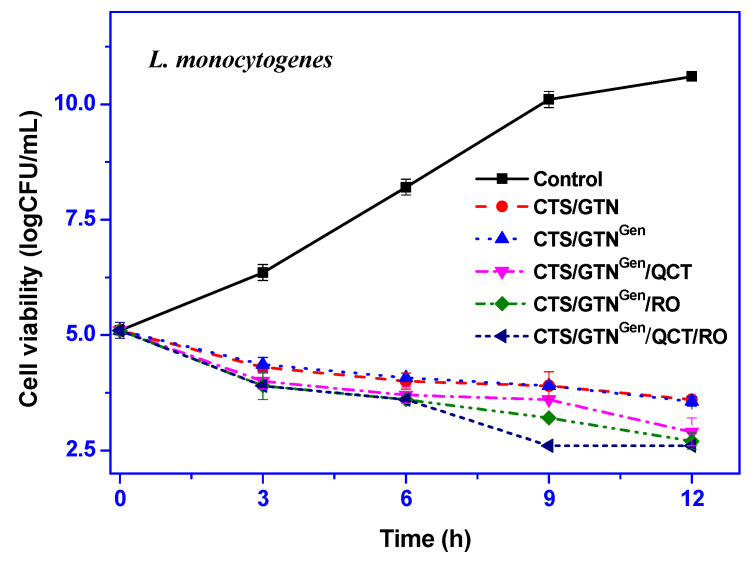
Antimicrobial activity of the chitosan/gelatin-based films against *E. coli* and *L. monocytogenes*.

The antimicrobial activity of the chitosan/gelatin-based films was tested against *E. coli* and *L. monocytogenes*, and the results are shown in Figure 4. The neat chitosan/gelatin film showed significant antibacterial activity against both Gram-negative and Gram-positive bacteria due to the antimicrobial functions of chitosan [49]. The antibacterial action of chitosan is thought to be due to the interaction between chitosan with polycations and bacterial cell membranes with polyanions [50]. 

Rosemary essential oil is also known for antimicrobial action, and the addition of rosemary oil in a packaging system showed significantly increased antimicrobial activity [34]. The addition of a crosslinker (genipin) did not affect the antibacterial function of the film. On the other hand, the antibacterial activity of the film was significantly increased by adding the functional fillers. However, the effect of the increase in the antibacterial activity depended on the type of functional fillers. The addition of quercetin slightly increased the antibacterial activity; however, the addition of rosemary essential oil increased the antibacterial activity more significantly. 

A slight antimicrobial activity of quercetin was noted in biopolymer films [20]. The combined addition of the functional fillers showed higher antimicrobial activity. Although the functional films showed significant antibacterial activity, they could not stop the growth of *E. coli* and *L. monocytogenes* completely. However, the functional films reduced the growth of *E. coli* and *L. monocytogenes* by 7.3–7.6 log CFU/mL and 6.8–7.7 log CFU/mL compared with the control group at 12 h of incubation.

The antibacterial activity of rosemary essential oil is attributed to its bioactive components, including rosemanol, epirosmanol, and rosmarinic acid [19]. Although there is no clear mechanism for antibacterial action, the bioactive components of essential oils have the effect of damaging cell membranes through the interaction between membrane proteins and essential oils [34]. In the case of quercetin, polyphenol flavonoids are mainly responsible for its antibacterial action [38]. The main target sites for flavonoid compounds are cell membranes, and their interaction is most likely to impair microbial growth by damaging the phospholipid bilayer [40].

### 3.3. Antioxidant Activity

**Figure 5:**
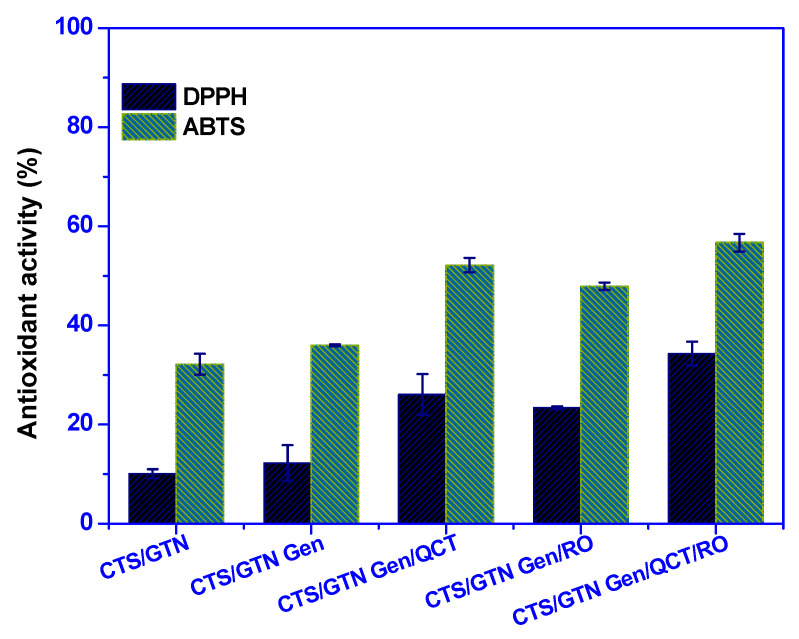
Antioxidant activity of the chitosan/gelatin-based films.

The antioxidant activity of the chitosan/gelatin-based films was also tested using the DPPH and ABTS methods, and the results are shown in Figure 5. The neat chitosan/gelatin film exhibited significant antioxidant activity derived from chitosan and gelatin. The antioxidant activity of chitosan is well known, and its function depends on functional groups, such as OH (C6) and NH_2_ (C2) [51]. Gelatin is also known to have antioxidant activity, which originates from the peptide bonds [52]. It is worth mentioning that the antioxidant action is superior against ABTS compared with DPPH due to the variable solubility of the hygroscopic biopolymers. 

Crosslinking by genipin did not significantly affect the antioxidant performance of the film. However, the addition of the functional fillers (quercetin and rosemary essential oil) significantly increased the antioxidant activity of the film. The combined addition of quercetin and rosemary essential oils increased the antioxidant further. The increment in antioxidant action is presumably owing to the antioxidant active materials, quercetin and rosemary essential oils. 

Previously, Yeddeds et al. also found that the antioxidant activity of gelatin-based films increased significantly with the addition of rosemary essential oil [31]. The antioxidant activity of rosemary essential oil comes primarily from polyphenols, such as rosemanol, epirosmanol, and rosmarinic acid. Quercetin is also known to have excellent antioxidant action due to the existence of polyphenolic flavonoid components. Yadav et al. also reported on the antioxidant activity of quercetin added to chitosan/gelatin films [20].

## 4. Conclusions

Genipin-crosslinked chitosan/gelatin-based functional films were prepared by adding quercetin (QR) and rosemary essential oil (RO). The blending of gelatin and chitosan resulted in highly transparent (light transmittance at 660 nm over 90%) and compatible films. The genipin-crosslinked chitosan/gelatin film completely blocked UV light, and the film’s strength was increased (10% increase in TS). The film’s water vapor barrier and surface wettability were not significantly affected by the addition of crosslinking agent and functional fillers. The addition of the functional fillers significantly increased the antioxidant and antimicrobial activity of the film. Therefore, the manufactured multifunctional chitosan/gelatin-based film has good prospects in active food packaging applications.

## Figures and Tables

**Table 1:** Surface color and light transmittance of the chitosan/gelatin-based films.

Films	*L*	*a*	*b*	Δ*E*	T_280_ (%)	T_660_ (%)
CTS/GTN	90.6 ± 0.2 ^b^	−1.1 ± 0.1 ^c^	8.1 ± 0.4 ^b^	4.0 ± 0.4 ^a^	21.00 ± 3.2 ^b^	91.0 ± 0.3 ^c^
CTS/GTN^Gen^	41.8 ± 2.7 ^b^	−5.1 ± 1.3 ^b^	−6.7 ± 0.7 ^a^	52.2 ± 2.5 ^b^	0.05 ± 0.0 ^b^	61.9 ± 2.3 ^b^
CTS/GTN^Gen^/QCT	31.3 ± 0.8 ^a^	−8.9 ± 0.7 ^a^	8.7 ± 0.7 ^b^	61.9 ± 0.6 ^c^	0.01 ± 0.0 ^a^	44.4 ± 1.0 ^a^
CTS/GTN^Gen^/RO	39.7 ± 1.3 ^a^	−4.1 ± 0.7 ^b^	−5.6 ± 0.3 ^a^	54.0 ± 1.2 ^b^	0.02 ± 0.0 ^a^	59.8 ± 1.3 ^b^
CTS/GTN^Gen^/QCT/RO	28.8 ± 0.7 ^a^	−6.5 ± 0.6 ^b^	7.4 ± 0.5 ^b^	64.1 ± 0.7 ^c^	0.01 ± 0.0 ^a^	39.2 ± 0.8 ^a^

Any two means in the same column followed by the same letter are not significantly (*p* > 0.05) different by Duncan’s multiple range test.

**Table 2:** The mechanical properties, water vapor permeability, and water contact angle of the chitosan/gelatin-based films.

Films	Thickness (μm)	TS (MPa)	EB (%)	EM (GPa)	WVP (×10^−9^ g·m/m^2^·Pa·s)	WCA (deg.)
CTS/GTN	44.3 ± 2.8 ^b^	77.3 ± 5.1 ^a^	6.3 ± 1.4 ^b^	3.2 ± 0.3 ^a^	0.65 ± 0.10 ^a^	60.5 ± 2.5 ^a^
CTS/GTN^Gen^	42.8 ± 2.5 ^a^	83.7 ± 3.9 ^b^	5.4 ± 0.6 ^a^	3.4 ± 0.2 ^b^	0.66 ± 0.06 ^a^	61.8 ± 2.6 ^a^
CTS/GTN^Gen^/QCT	42.5 ± 2.4 ^b^	84.8 ± 5.0 ^b^	5.4 ± 0.4 ^a^	3.4 ± 0.2 ^b^	0.69 ± 0.03 ^a^	60.9 ± 1.5 ^a^
CTS/GTN^Gen^/RO	43.8 ± 3.7 ^b^	84.8 ± 4.3 ^b^	5.5 ± 0.6 ^a^	3.4 ± 0.2 ^b^	0.67 ± 0.07 ^a^	60.3 ± 1.5 ^a^
CTS/GTN^Gen^/QCT/RO	44.7 ± 2.8 ^b^	86.7 ± 5.0 ^c^	5.7 ± 0.4 ^a^	3.4 ± 0.2 ^b^	0.71 ± 0.09 ^b^	61.5 ± 1.6 ^a^

Any two means in the same column followed by the same letter are not significantly (*p* > 0.05) different by Duncan’s multiple range test.

## Data Availability

Not applicable.

## Supplementary Materials

The following supporting information can be downloaded at: https://www.mdpi.com/article/10.3390/ma15113769/s1, Figure S1: FTIR spectral patterns of the chitosan/gelatin-based films; Table S1: Thermogravimetric analyzed data of the chitosan/gelatin-based films. References [53,54,55,56] are cited in the Supplementary Materials.
