# Supplementary material for: Genipin-Crosslinked Gelatin/Chitosan-Based Functional Films Incorporated with Rosemary Essential Oil and Quercetin
Source: Materials (Basel). 2022 May 25;15(11):3769. doi: 10.3390/ma15113769 (PMC9181465; doi:10.3390/ma15113769)
Supplement: Supplementary file 1 [file materials-15-03769-s001.zip › materials-1716021-supplementary.pdf]

materials

MDPI

Supplementary files

Genipin-Crosslinked Gelatin/Chitosan-Based Functional Films Incorporated with Rosemary Essential Oil and Quercetin

Swarup Roy [1,2] and Jong-Whan Rhim [1,*]

[1] Department of Food and Nutrition, BioNanocomposite Research Institute, Kyung Hee University, 26 Kyungheedae-ro, Dongdaemun-gu, Seoul 02447, Korea; swaruproy2013@gmail.com
[2] School of Bioengineering and Food Technology, Shoolini University, Solan 173229, Himachal Pradesh, India
* Correspondence: jwrhim@khu.ac.kr

Citation: Roy, S.; Rhim, J.-W. Genipin-Crosslinked Gelatin/Chitosan-Based Functional Films Incorporated with Rosemary Essential Oil and Quercetin. *Materials* **2022**, *15*, x. https://doi.org/10.3390/xxxxx

Academic Editor: Luc Avérous

Received: 22 April 2022
Accepted: 23 May 2022
Published: date

Publisher's Note: MDPI stays neutral with regard to jurisdictional claims in published maps and institutional affiliations.

Copyright: © 2022 by the authors. Submitted for possible open access publication under the terms and conditions of the Creative Commons Attribution (CC BY) license (https://creativecommons.org/licenses/by/4.0/).

1. Materials and Methods

Materials

Food grade gelatin (Type A, 200 Bloom) was procured from Gel-Tec Co. Ltd. (Seoul, Korea). Chitosan (200-800 cP, MW: 190,000-310,000, 75–85% deacetylated), ABTS, DPPH, genipin, quercetin, and potassium persulfate were bought from Sigma-Aldrich (St. Louis, MO, USA). Rosemary essential oil (100% pure) was procured from JK Group Co., Ltd. (Bucheon, Gyeonggi-do, Korea).

2. Characterization and Properties of the Film

2.1. Surface Color and Optical Properties

The Hunter color (L, a, and b) values of the film sample were also measured using a Chroma meter (Konica Minolta, CR-400, Tokyo, Japan) with a white standard plate as a background. The total color difference (ΔE) was calculated as follows:

$$\Delta E = \sqrt{(\Delta L)^2 + (\Delta a)^2 + (\Delta b)^2} \quad (1)$$

where ΔL, Δa, and Δb were differences between each color value of the control film and the test film.

The light transmittance spectra of all the films were noted using a UV-vis spectrophotometer (Mecasys Optizen POP Series UV/Vis, Seoul, Korea). The UV-barrier and transparency properties of the film were also evaluated by measuring the percent transmittance of the film sample (5 × 5 cm) at 280 nm (T_{280}) and 660 nm (T_{660}), respectively [53].

2.2. Morphology and FTIR

The films' surface and cross-sectional morphology were observed using a field emission scanning electron microscope (FE-SEM, SU 8010, Hitachi Co., Ltd., Matsuda, Japan) at an accelerating voltage of 1 kV. Film specimens were vacuum sputter-coated with platinum for 120 s before testing.

FTIR spectra of the film samples specimen were recorded using a Fourier transform infrared spectrometer (TENSOR 37 Spectrophotometer with OPUS 6.0 software, Billerica, MA, USA) in attenuated total reflection (ATR) mode with the wavenumber ranging from 4000–650 cm^{-1} at 32 scan rate with the resolution of 4 cm^{-1}.

2.3. Mechanical Properties

The film's thickness was measured using a digital micrometer (Digimatic Micrometer, QuantuMike IP 65, Mitutoyo, Japan) with an accuracy of 1 μm. The mechanical properties such as tensile strength (TS), elongation at break (EB), and elastic modulus (EM) of the film were determined according to the standard method of ASTM D 882-88 using an Instron Universal Testing Machine (Model 5565, Instron Engineering Corporation, Canton, MA, USA). The Instron machine was operated with an initial grip separation of 50 mm and a crosshead speed of 50 mm/min [52].

2.4. Water Vapor Permeability (WVP) and Water Contact Angle (WCA)

The WVP of the film was measured gravimetrically using a WVP cup according to the ASTM E96-95 standard method. The WVP cup was filled with 18 mL of distilled water and then covered with film, sealed tightly, and kept in the controlled environmental chamber controlled at 25 °C and 50% RH. After equilibration, the weight of the WVP cup was measured at every one-hour interval, and the weight loss was calculated. The WVTR (g/m^2.s) was determined from the slope (linear) of the steady-state portion of weight loss of the cup versus the time curve. Then, the WVP (g.m/m^2.Pa.s) of the film was calculated as follows:

$$WVP = (WVTR \times L)/\Delta p \quad (2)$$

where L was the thickness of the film (m), and Δp was water vapor partial pressure difference (Pa) across the film.

The film's surface wettability was evaluated by measuring the water contact angle of the film surface using a WCA analyzer (Phoneix 150, Surface Electro Optics Co., Ltd., Kunpo, Korea). The film sample was fixed on the film holder, and a drop of water (~10 μL) was added to the film's surface and measured the WCA.

2.5. Thermal Stability

The thermal stability of the film sample was evaluated using a thermogravimetric analyzer (Hi-Res TGA 2950, TA Instrument, New Castle, DE, USA). For this, ~10 mg of film sample was taken in a standard aluminum pan and scanned at a heating rate of 10 °C/min in a temperature range of 30–600 °C under a nitrogen flow of 50 cm^3/min [23].

2.6. Antibacterial and Antioxidant Activity

The antibacterial activity of the films was determined using a total viable colony count method [52]. The food-borne pathogenic bacteria, *L. monocytogenes* and *E. coli,* were used for this study. Thus, *L. monocytogenes* and *E. coli* were inoculated in the BHI and TSB broth, respectively, and then cultured overnight at 37 °C with gentle shaking. The next day, the inoculum was appropriately diluted, and 100 μL was aseptically transferred to 20 mL of TSB and BHI broth (10^4 and 10^5 CFU/mL) containing 150 mg of the film sample and incubated at 37 °C for 12 h with agitation at 100 rpm. Samples were taken at a predetermined time interval and plated on agar plates after appropriate dilution to assess the viable colony count. For evaluation, antibacterial tests were performed using film-free culture media and the neat film as negative and positive controls.

Antioxidant activities of the films were assessed using 2,2-diphenyl-1-picrylhydrazyl radical (DPPH$^•$) and 2,2′-azino-bis(3-ethylbenzothiazoline-6-sulfonic acid) (ABTS$^{•+}$) radical scavenging methods [54]. For DPPH analysis, a new methanolic solution of DPPH was prepared, and ~100 mg of the tested film sample was added to a 20 mL DPPH solution, incubated for 360 min at room temperature. We measured the absorbance at 517 nm. For ABTS analysis, potassium sulfate (2.45 mM) was mixed with ABTS solution (7 mM) and incubated overnight in the dark to produce an ABTS assay solution. One hundred mg of film samples were mixed in 20 mL of ABTS assay solution and incubated at room temperature for 30 min in the dark, and then the absorbance was measured at 734 nm. The antioxidant activity of all the films was determined as follows:

$$\text{Free radical scavenging activity (\%)} = \frac{A_c - A_s}{A_c} \times 100 \quad (3)$$

where A_c and A_s were the absorbance of DPPH and ABTS of the control and test films, respectively.

3. Statistical Analysis

One-way analysis of variance (ANOVA) was performed, and the significance of each mean property value was determined ($p < 0.05$) with Duncan's multiple range test using the SPSS statistical analysis computer program for Windows (SPSS Inc., USA).

4. Results

FTIR Analysis

The FTIR spectra of all the fabricated films are shown in Figure S1. At 3290 cm^{-1}, the broad peak appeared mainly owing to O-H stretching [20]. The peaks detected at 2939 and 2878 cm^{-1} were likely due to the C-H stretching vibrations [55]. The 1631 and 1545 cm^{-1} peaks were due to the C=O and N-H stretching vibrations [20]. The 1452, 1406, and 1239 cm^{-1} peaks were recognized as C–N and N–H stretching, O-H bending, and amide-III of the gelatin, respectively [52]. The peaks at 1150 and 1027 cm^{-1} were accredited to the saccharide structure and C=O stretching vibrations [56]. In the case of cross-linker and bioactive filler incorporated films, some minor variations in the peak position and intensity were noticed; otherwise, the pattern was similar to the neat chitosan/gelatin film. The slight changes in the chemical characteristics of the film could be due to the physical interactions among the polymers and fillers.

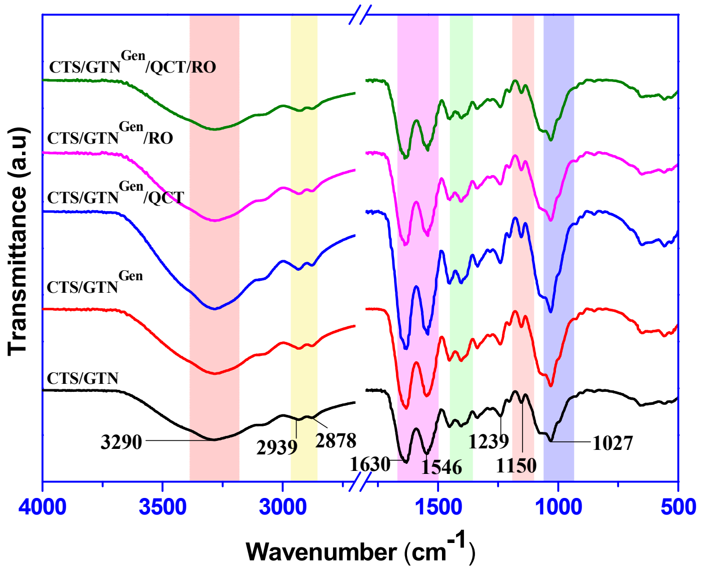

Figure S1. FTIR spectral patterns of the chitosan/gelatin-based films.

Table S1 (Supplementary Materials) shows more detailed TGA analysis results.

Table S1. Thermogravimetric analyzed data of the chitosan/gelatin-based films.

Films	T_{onset}/T_{end} (°C)	$T_{0.5}$ (°C)	Char at 600 °C (%)	T_{max} (°C)
CTS/GTN	146/465	356	36.2	298
CTS/GTN^{Gen}	144/495	362	36.2	310
$CTS/GTN^{Gen}/QCT$	144/495	366	38.2	309
$CTS/GTN^{Gen}/RO$	145/496	363	36.3	309
$CTS/GTN^{Gen}/QCT/RO$	145/495	365	36.8	310
